# Tetracyclines, a timely resurgence or doomed to fail again?

**DOI:** 10.1099/jmm.0.002197

**Published:** 2026-09-04

**Authors:** Samuel Fenn, Dimitra Panagiotopoulou, Kim R. Hardie

**Affiliations:** 1 School of Life Sciences, The Biodiscovery Institute, University of Nottingham, Nottingham, UK; 2 National Biofilms Innovation Centre, The Biodiscovery Institute, University of Nottingham, Nottingham, UK; 3 School of Microbiology, University College Cork, Cork, Ireland; 4 APC Microbiome, University College Cork, Cork, Ireland

**Keywords:** antibiotic, AMR, antimicrobial resistance, tetracycline

## Abstract

Tetracyclines are bacteriostatic antimicrobials used in the treatment of both bacterial and protozoan pathogens. They abolish protein synthesis by binding to the 16S rRNA, blocking aminoacyl-tRNA access and preventing polypeptide elongation. Tetracyclines are polyketide antimicrobials, with members of this drug family (doxycycline, minocycline and tigecycline) on the World Health Organization (WHO) list of essential medicines. Bacterial resistance to this antimicrobial family is widespread and arises via multiple mechanisms. The most common of these resistance mechanisms are tetracycline efflux, ribosomal protection proteins and the newly emerging tetracycline destructases (TDases). The rise of resistance has limited the clinical use of first- and second-generation tetracyclines; however, third-generation tetracyclines are now reserved as antimicrobials of last resort for complicated bacterial infections. However, genetically mobile TDases have now been identified in opportunistic pathogens, with this mechanism of resistance capable of inactivating third-generation tetracyclines. This presents the question, are third-generation tetracyclines doomed to fail in the same manner as first-generation tetracyclines?

## Graphical Abstract

Brief diagrammatic overview of tetracyclines structure, mode of action, side-effects, spectrum of action and administration. Tetracyclines are type II polyketides consisting of four fused rings, with an oxygen-rich ‘non-modifiable’ region required to maintain antimicrobial activity. Tetracyclines target the prokaryotic ribosome by binding the 30S subunit, inhibiting polypeptide elongation. These antimicrobials have a broad spectrum of activity targeting Gram-positive and Gram-negative bacteria alongside eukaryotic parasites. Tetracyclines are administered both orally and intravenously and cause a wide range of side effects, primarily driven by the ability of tetracycline to chelate essential metal ions and bind to the mitochondrial ribosomes. The graphics were created in BioRender. Fenn, S. (2026) https://BioRender.com/fdhfh5s

## Historical perspective

Tetracyclines were isolated from soil *Actinomycetes* by Lederle laboratories and first utilized clinically in 1948 [1]. This antimicrobial inhibits protein synthesis, binding to the 16S subunit of the ribosomes in both bacteria and the mitochondria of a limited number of parasites, including *Plasmodium spp*. (malaria) and *Toxoplasma gondii* [1–3]. First-generation tetracyclines are naturally produced compounds, including chlortetracycline, oxytetracycline and tetracycline. In subsequent years, the core structure of tetracycline was modified with functional groups to improve the ADMET profile of these drugs (adsorption, distribution, metabolism, excretion and toxicity) and overcome widespread drug resistance. Second-generation tetracyclines doxycycline and minocycline exhibited enhanced pharmacokinetic properties against a broad range of bacteria and received Food and Drug Administration (FDA) approval in 1967 and 1971, respectively [4]. Recently developed third-generation tetracyclines were specifically designed to overcome common tetracycline resistance mechanisms [5, 6]. These include tigecycline (FDA-approved 2005), eravacycline, omadacycline and sarecycline (all FDA-approved 2018) [5, 7–9]. This development of new generations of tetracyclines has consistently been followed by the emergence of novel tetracycline-resistance mechanisms (discussed in resistance mechanisms). This trend has seen tigecycline being reserved as an antibiotic of last resort in multidrug-resistant infections to prevent widespread usage and slow down the development of resistance.

## Structure/chemistry

Tetracyclines are type II polyketides composed of four (tetra) linearly fused carbocyclic rings (cyclines), with this base structure known as a naphthacene core. The antibacterial activity requires conservation of ribosomal binding, with the lower non-modifiable region essential for this interaction. The conserved domain consists of the naphthacene core with a C1–C3 keto-enolic tautomer, C2-amide, C4-dimethylamino, C10-phenol, C11–12 keto-enol substructure and C12-hydroxyl group [10].

Chemical modification of tetracyclines primarily occurs at five positions (C5–C9) on the B, C and D rings in the upper modifiable region, resulting in production of the second- and third-generation tetracyclines [10]. Attachment of different functional groups at these positions are responsible for the enhanced pharmacological and drug-resistant properties of these tetracyclines. For example, addition of a hydroxyl group at C5 and removal of a hydroxyl group at the C6 position results in the production of the second-generation tetracycline doxycycline [11]. The addition of a 5-hydroxyl group increases acid stability of doxycycline, increasing the half-life from 6 to 12 to 15–25 h when administered orally. Whilst the removal of the 6-hydroxyl group increases the lipophilicity of doxycycline, promoting increased tissue penetration [11, 12]. These substitutions combined improve the bioavailability of doxycycline and reduce the number of doses administered daily (2 vs 4) as compared to first-generation tetracycline. Third-generation tetracyclines contain bulky substitutions at the C9 position. For example, tigecycline contains a 9-*t*-butylglycylamido moiety which enables this third-generation tetracycline to evade clinically relevant resistance mechanisms, such as efflux pumps [5]. Similarly, eravacycline contains a 9-pyrrolidinoacetamido group, with omadacycline utilizing a 9-aminomethyl moiety, which enables these third-generation antimicrobials to maintain activity in the presence of widely distributed resistance mechanisms [7, 9].

## Mode of action

**Fig. 1.**
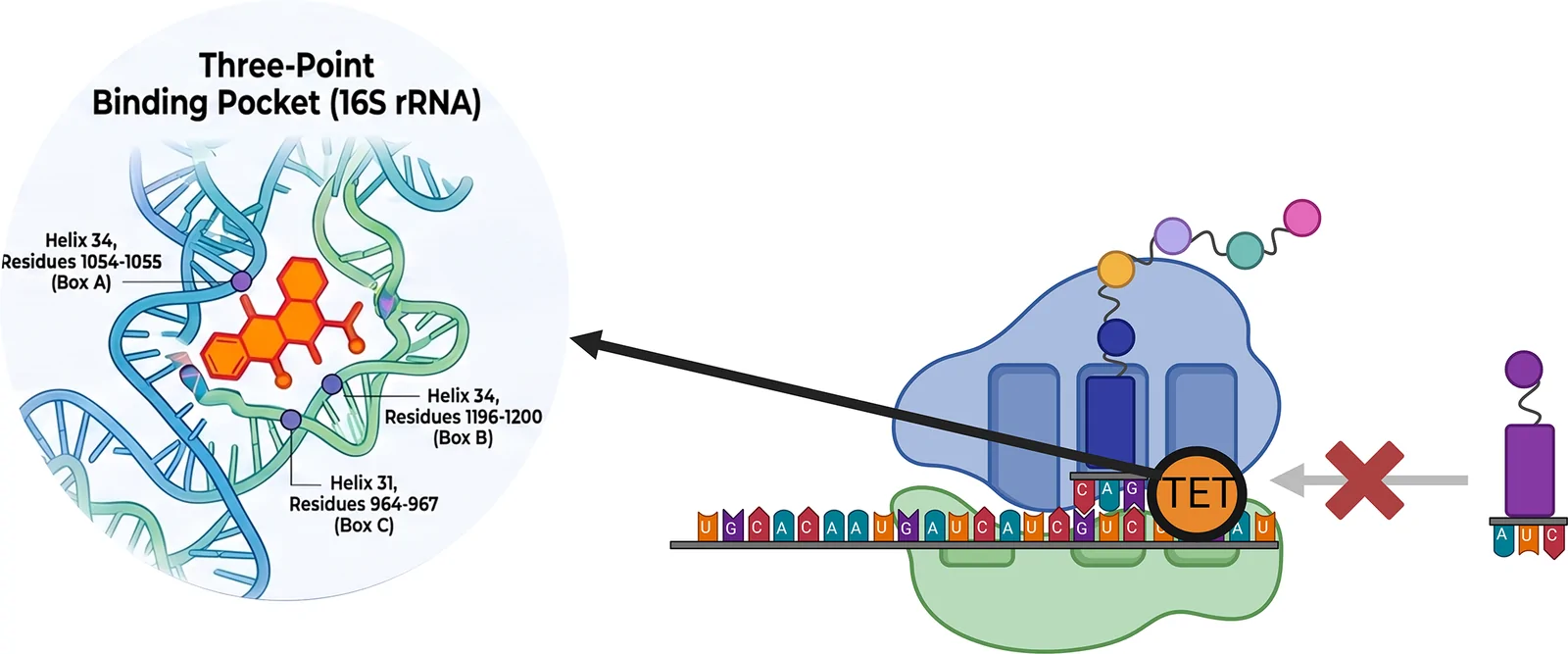
Tetracycline mode of action. Tetracycline binds to 16S rRNA of the 30S prokaryotic ribosome. Tetracycline binding is dependent on three key boxes located on Helices 31 and 34, with specific RNA residues essential for localization of tetracycline at the aminoacyl delivery site. This positioning prevents new tRNAs and aa from entering the aminoacyl site, blocking elongation of the polypeptide chain. Created in BioRender. Fenn, S. (2026) https://BioRender.com/6hfwwiu.

Tetracyclines are bacteriostatic antimicrobials which bind to the 30S ribosomal subunit of bacteria and interact with a highly conserved 16S rRNA region. The binding pocket consists of rRNA residues 1,054–1,055 (box A), 1,196–1,200 (box B) on helix 34 and 964–967 of helix 31 (box C) (Fig. 1) [13, 14]. These interactions are primarily with the sugar-phosphate backbone of the rRNA, with binding in this region sterically blocking docking of tRNAs at the aminoacyl site (A-site) [13]. This inhibits polypeptide elongation and prematurely terminates protein translation (Fig. 1). Both humans and bacteria possess ribosomes; however, eukaryotic ribosomes are larger (40S and 60S) and have structural differences in the small ribosome rRNA subunit, meaning tetracyclines bind with a 15-fold reduced affinity when compared to prokaryotic ribosomes [15].

Eukaryotic mitochondria are descendants of modern prokaryotes and possess a similar ribosomal structure. Tetracyclines can negatively impact protein translation in mitochondria at high concentrations, impairing the ability of eukaryotes to generate ATP. This property has been exploited in the treatment of parasites, such as *Plasmodium spp*. (malaria) and *T. gondii* (toxoplasmosis), with minocycline and doxycycline used as adjunctive therapies to treat these infections [2, 3]. Tetracyclines selectively target these parasites through inhibition of mitochondria in essential specialized organelles called apicoplasts [2, 16]. These membrane-bound organelles host essential metabolic pathways, such as fatty acid, isoprenoid and haem synthesis, with these organelles not present in human cells providing selectivity when used for the treatment of humans harbouring these eukaryotic pathogens [2, 16]. Mitochondrial ribosomal structure has also diverged from prokaryotes, with tetracyclines demonstrating reduced affinity for mitochondrial ribosomes in comparison to prokaryotes.

## Cellular entry

Passage of tetracyclines into the bacterial/mitochondrial cytoplasm is dependent on the membrane structure of the target organisms. To cross the Gram-negative and mitochondrial outer membrane, tetracyclines chelate divalent cations, such as Mg^2+^, with this charged tetracycline–metal complex passively diffusing through water-filled membrane porin channels (OmpF and OmpC) [17]. Once in the periplasm of Gram-negatives or in the extracellular space of Gram-positives, the tetracycline–metal complex dissociates. Subsequently, the lipophilic tetracycline is able to pass through the cytoplasmic membrane by passive diffusion [17, 18]. Tetracycline crossing of the cytoplasmic membrane has also been linked to a ∆pH-dependent process which relies on the accumulation of protons and formation of a proton–tetracycline complex, increasing cytoplasmic membrane permeability of the drug [18]. Once in the cytoplasm, tetracycline binding to the 30S ribosome is dependent on forming a complex with Mg^2+^ again. This metal ion is required to effectively bind the sugar-phosphate backbone of the conserved 16S rRNA tetracycline binding site [19, 20]. Tetracyclines also chelate other divalent cations, such as Ca^2+^ and Fe^2+^ [21, 22]. Binding to these cations results in the formation of an insoluble and inactive metal–tetracycline complex which reduces the ability of the human body to absorb the antibiotic. This has a significant impact on tetracycline activity, with administration of this drug most effective when taken on an empty stomach [22].

The lipophilicity of the tetracycline dictates the ability of these drugs to cross the cytoplasmic membrane and accumulate within the cytoplasm compartment of bacteria. For example, a C6 substitution of a hydroxyl group with a non-polar methyl group in doxycycline drastically increases the lipophilicity of doxycycline in comparison to first-generation tetracyclines. As a result, doxycycline accumulates in the bacterial cytoplasm more efficiently, alongside demonstrating increased absorption by the human body. This property means doxycycline is the tetracycline of choice for treating intracellular parasites, as it efficiently crosses the membrane of human cells.

## Resistance mechanisms

Tetracycline resistance is widespread amongst bacteria, with new resistance mechanisms quickly emerging following the development of each tetracycline generation [23]. A range of mechanisms have evolved to inhibit the action of tetracyclines; however, they are centred around three key methods: (i) remove/prevent the antibiotic binding the target; (ii) antimicrobial target modification and (iii) permanently inactive the antibiotic.

### Remove/Prevent tetracycline binding to ribosome

Removing or preventing tetracycline binding to ribosomes reduces the intracellular concentration of tetracycline, decreasing association of this antibiotic with the ribosome enabling protein synthesis to continue. The primary mechanisms by which this occurs in bacteria are through removal of tetracycline from the cytoplasm with dedicated transporters; expression of ribosomal protection proteins (RPPs) which displace tetracycline bound to ribosomes; and bacterial membrane permeability modifications which occlude tetracycline from the cytoplasm [24].

#### Efflux pump-mediated tetracycline resistance

**Fig. 2.**
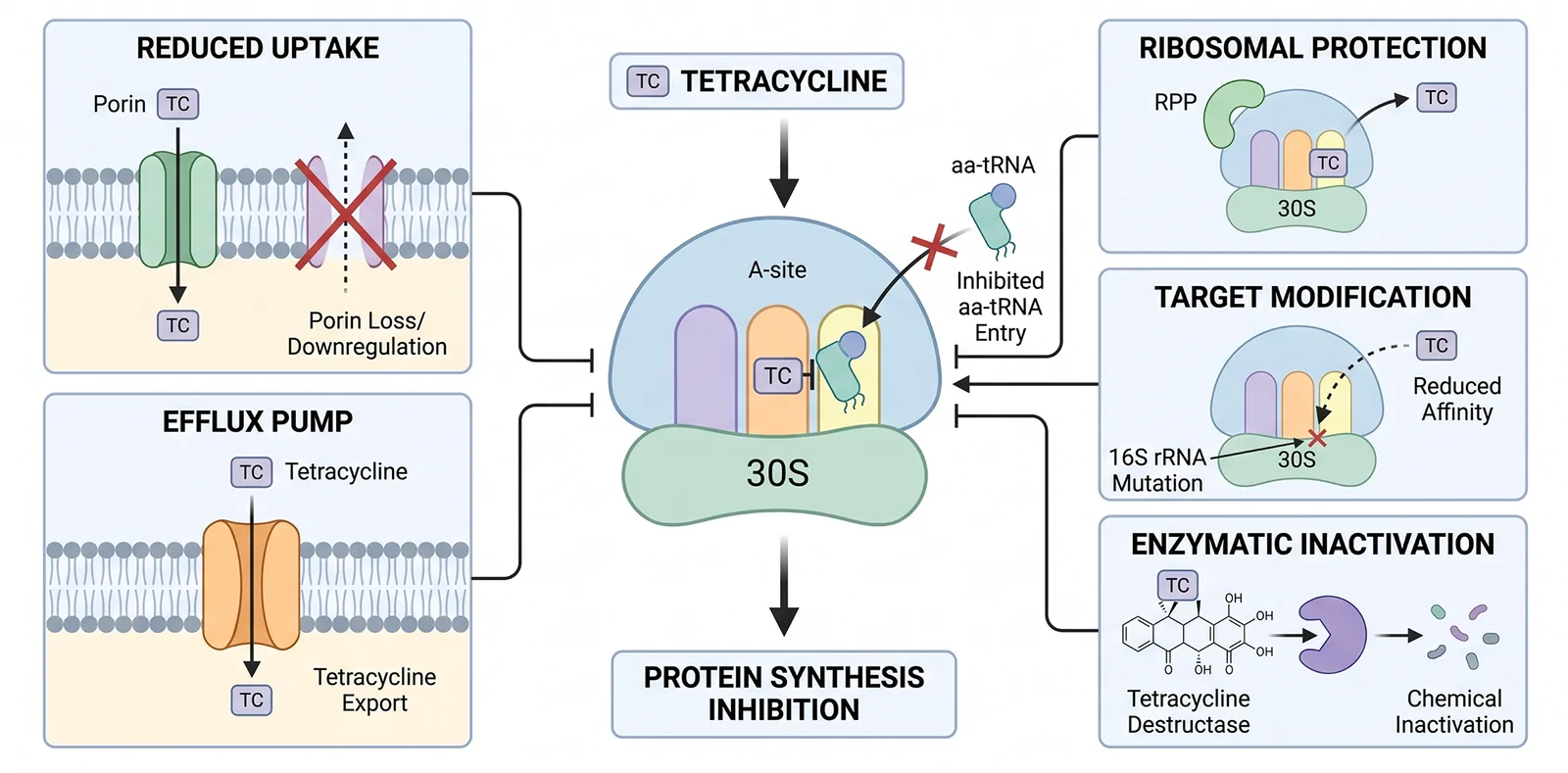
Tetracycline: mechanism of action and resistance. Upon entry, tetracyclines bind with the 16S rRNA of the 30S ribosomal subunit. This interaction prevents elongation of the protein polypeptide chain by sterically hindering access of aminoacyl tRNAs. Tetracycline resistance is widespread amongst Gram-positive and Gram-negative bacteria. It arises through five main mechanisms: (i) efflux removal of tetracycline from the cytosol and target site; (ii) RPP-mediated alteration of ribosome–tetracycline interactions; (iii) modified outer membrane and porin protein expression reducing permeability; (iv) ribosomal mutations in the 16S rRNA of the 30S subunit reduce affinity of tetracycline for the ribosome; (v) tetracycline destructases (TDases) chemically inactivate the naphthacene core of tetracyclines abolishing antimicrobial activity. Created in BioRender. Fenn, S. (2026) https://BioRender.com/fdhfh5s.

Efflux pumps are cytoplasmic membrane-associated transporters which actively pump the drug out of the cytoplasm as a metal–tetracycline-bound chelate [17, 25]. These efflux systems are divided into two main categories based on substrate specificity: (i) tetracycline-specific transporters and (ii) multidrug-resistance (MDR) pumps. Both systems function as antiporters and require energy through proton-motive force to function, with a proton pumped into the cytoplasm whilst tetracycline is pumped out (Fig. 2a). Tetracycline-specific transporters typically belong to the Major Facilitator Superfamily (MFS) with 12–14 transmembrane domains.

Efflux is mediated by 25 distinct *tet* genes with *tetA*, *tetB* and *tetC* common in Gram-negatives, whilst *tetK* and *tetL* are associated with Gram-positives [23]. MDR pumps, such as the prototypical AcrAB–TolC system, can transport a broad range of substrates, including tetracycline [26]. These pumps belong to the Resistance-Nodulation-Cell-Division (RND) family and are generally associated with Gram-negative bacteria, where this system forms a tripartite pump which spans the inner membrane, periplasm and outer membrane enabling direct extrusion of antibiotics extracellularly [26]. Tetracycline-specific efflux pumps are responsible for high-level resistance against first-generation tetracyclines, in contrast MDR pumps generally result in lower levels of resistance. For example, production of TetA in *Escherichia coli* increased the MIC of oxytetracycline up to 128–512 mg l^−1^ whilst AcrAB–TolC (RND) increased resistance to 4–64 mg l^−1^ [27, 28]. One advantage MDR pumps have over specific transporters is the ability to expel all generations of tetracycline irrespective of modifications [29]. The second- and third-generation modifications of tetracyclines mean these variants do not serve as substrates for MFS tetracycline-specific efflux pumps [30]. One notable exception to this is TetB which can efflux second-generation tetracyclines [31]; however, third-generation variants are not substrates for this pump due to the bulky modifications at the C9 position.

#### Ribosomal protection proteins

RPPs directly sense ribosomal stalling following tetracycline binding and physically insert into the ribosome. This drives a conformational change in the structure of the ribosome and tetracycline binding site which enables RPP to act as a molecular ‘crowbar’ and displace tetracycline from the aminoacyl acceptor site (Fig. 2b) [32, 33]. RPPs are structurally related to elongation factor (EF-G) and elongation factor Tu (EF-Tu), prokaryotic GTPases involved with tRNA binding and delivery to the ribosome. This shared ancestry enables RPPs to bind to ribosomes at the aminoacyl site, with GTP hydrolysis causing a protein loop from the S10 ribosomal protein (loop III) to project into the tetracycline binding pocket, physically removing the antibiotic [33]. A functional elongation complex can then reform, enabling bacterial protein translation to proceed. RPPs are active against first- and second-generation tetracyclines, with this resistance mechanism first reported in 1980 shortly after clinical use of the second-generation tetracyclines minocycline and doxycycline [23, 34]. Third-generation tetracyclines were specifically designed to counteract this form of resistance, with C9 modifications inhibiting RPP binding to the S10 ribosomal protein [35].

#### Limitation of tetracycline influx

Reduction of tetracycline entry to the cytoplasm is a mechanism of resistance primarily associated with Gram-negative bacteria through modification of the outer membrane. Tetracyclines gain access to the periplasm of Gram-negatives through outer membrane porins, such as OmpF and OmpC (Fig. 2c) [36]. Decreased porin expression, complete loss of porins or alterations in the electrostatic charge within porins have been shown to drive increased resistance to tetracyclines (Fig. 2c). Antibiotic stress leads to an adaptive response, with activation of the *marRAB* operon in Gram-negatives leading to upregulation of the small RNA *micF*. This small RNA binds to *ompF* mRNA leading to downregulation of this porin, reducing the rate of tetracycline entry into the cytoplasm [37, 38]. Similarly, complete loss of OmpF or OmpC through frame-shift mutations is a common mechanism reported to increase Gram-negative resistance to tetracyclines by limiting ingress. However, since essential nutrients, such as sugars, aas and ions also use these porins to gain access to the cell, inactivating mutations of these porins are associated with decreased fitness in the absence of antibiotic pressure [39]. aa substitutions which modify the internal electrostatic charge of the porin to reduce transit of large molecules, such as tetracyclines have been identified in clinical isolates. These mutations still allowed transport of essential nutrients and ions, however, at reduced abundances, the mutated alleles still imparted a growth defect in *E. coli* [40]. These mechanisms increase the intrinsic resistance of Gram-negative bacteria to multiple antibiotics. Alone, they only provide limited resistance. Often these modifications operate alongside a synergistic upregulation of efflux, with the MarR regulator also demonstrated to increase expression of the *acrAB–tolC* system [28, 37]. Together, limited ingress of tetracycline combined with an improved ability to export tetracyclines contributed to clinically relevant increases in tetracycline resistance.

### Ribosomal modification

Target site modification is a common mechanism of antimicrobial resistance in bacteria. Modification of ribosomal structure is a commonly detected tetracycline resistance strategy, with most identified changes to the 16S rRNA and the S10 ribosomal protein associated with enhanced resistance (Fig. 2d). Both 16S rRNA and S10 mutations decrease the affinity of tetracyclines binding to the ribosome through altering helix 31 and helix 34. Both helices are essential ribosomal structures recognized and bound by tetracyclines [14, 35]. Base mutations in helix 31 (965–967) and helix 34 (1,058) of the 16S RNA sequence alter the structure of the RNA, thereby inhibiting tetracycline binding [14, 41]. The S10 ribosomal protein directly interacts with helix 31 and helix 34 of the 16S RNA, with aa changes altering the structure of tetracycline binding site and reducing binding affinity [35]. These mutations can provide resistance to all tetracycline generations, limiting the effectiveness of antibiotics of last resort, such as tigecycline [42]. Given that the ribosome is such an essential structure, modifications in structure are poorly tolerated. Mutations identified in *E. coli* associated with enhanced tigecycline resistance decreased the fitness of these clinical strains, manifested by decreased growth rates, increased thermosensitivity and reduced virulence [43]. What is not clear is how quickly these mutations can revert in the absence of the tetracyclines. Loss of selection pressure often results in restoration of WT function, especially when mutations are associated with a high fitness cost as is the case for ribosomal mutations [44]. This strategy enables a sub-population of tetracycline-resistant cells to survive treatment, with subsequent reversion enabling infections to take hold again in a ‘bet-hedging’ strategy employed by bacteria in response to many antibiotics.

### Tetracycline inactivation

Chemical modification of antimicrobials is a common mechanism of resistance in bacteria. Tetracycline resistance has classically been dominated by protective mechanisms, with continuous expression of efflux and RPPs required to maintain resistance in the presence of tetracycline. Tetracycline destructases (TDases) are a novel family of flavin monooxygenases capable of inactivating tetracyclines, directly reducing the amount of active antibiotic in the environment (Fig. 2e) [45]. This method of resistance was originally identified in 1988, with the *tetX* encoded on transposons in anaerobic *Bacteroides spp*. [45, 46]. Initially, identification of these sequences was rare, leading researchers to conclude that this mechanism of resistance was of low concern. However, the advent of the genomics age led to the identification of multiple TDases, with this family of enzymes globally distributed in human and environmental samples [47].

TDases are divided into two types based on sequence similarity and activity profiles. Type 1 TDases, including the originally identified TetX, are found in human gut isolates, microbiomes and opportunistic pathogens [47, 48]. Type 2 TDases are largely confined to soil isolates and only identified through functional metagenomics [48]. aa homology is limited between type 1 and 2 TDases, although all TDases share a similar structure which enables destruction of tetracyclines. TDases are a novel family of flavin monooxygenases which hydroxylates the naphthacene core structure at position C11a, initiating ring opening and degradation [48, 49]. Type 1 TDases are an emerging clinical concern, with these enzymes capable of inactivating all generations of tetracyclines. Similarly, type 1 TDases have been identified to be associated with multidrug-resistant pathogens, such as *Klebsiella pneumoniae*, *Pseudomonas aeruginosa and E. coli*. In contrast, type 2 TDases only inactivate first- and second-generation tetracyclines, and the lack of association with human commensal/pathogenic bacterial isolates limits the clinical impact of this destructase type [48]. There are now over 20 type 1 TDases, with many of these variants identified on transposons and plasmids alongside resistance genes which inactivate carbapenems and colistin, other antibiotics of last resort [23, 50–55]. Whilst efflux and RPPs are still the most common mechanisms of resistance identified in clinical strains, the presence of type 1 TDases is more dangerous clinically as treatment options are severely limited because third-generation tetracyclines act as substrates.

## Incidence and global dissemination of tetracycline resistance

Tetracycline resistance mechanisms predate widespread medical and animal husbandry use, with production by environmental bacteria necessitating mechanisms of resistance to compete [56]. The intense usage of tetracyclines in both the clinic and animal husbandry has driven an increase in the number of bacteria which encode one or more tetracycline-resistant mechanisms [23, 57]. This is caveated by the fact that improvements in detection through extensive genome sequencing and intense research into antimicrobial resistance mean that exact comparisons between years are difficult. However, the extensive diversification of resistance variants with over 30 *tet* efflux systems, 14 RPPs and 30 TDases indicates an ever-growing and evolving problem. Whilst resistance to tetracyclines is a global problem, the burden of resistance is dependent on multiple factors, including the country of isolation, the bacterial species and if the bacterial isolates are of human, animal or environmental origin [57].

The Tigecycline Evaluation Surveillance Trial (T.E.S.T.) study conducted between 2003 and 2016 measured *in vitro* resistance to tigecycline at a global scale, with all strains derived from human infections. Overall, resistant pathogens increased over time and levels of resistance varied across different regions. Minocycline resistance in *Enterobacteriaceae* was 25.1%, peaking in Latin America at 40% whereas only 12% of MDR *Enterobacteriaceae* strains were susceptible. *E. coli* exhibited resistance in 13–14% of isolates, with this increasing in extended spectrum beta-lactamase producing (ESBL)-producing *E. coli* to 22.7% [58, 59]. In this study, it was noted that resistance to the third-generation tetracycline tigecycline was non-existent in Gram-positives, whilst resistance was only detected in three Gram-negatives, *Enterobacter spp*. (5.2%), *K. pneumoniae* (5%) and *E. coli* (0.1%) [58, 59]. The T.E.S.T. study is now over a decade old, and the fast-evolving nature of antimicrobial resistance (AMR) means these numbers are not reflective of the modern day. Current monitoring by the European Centre for Disease Prevention and Control indicates that tetracycline resistance is endemic in Europe, with resistance rates amongst indicator bacteria increasing. For example, around 25–30% of *E. coli* isolates derived from humans are tetracycline-resistant in the European Union (EU), whilst ESBL-producing *E. coli* isolates now demonstrate resistance to tetracyclines in 66.7% of cases [60]. Whilst first- and second-generation tetracycline resistance appears to be increasing, tigecycline resistance appears to be low, with resistance detected in 1–2% of cases. This burden of tetracycline resistance is markedly increased in isolates of animal origin, with *E. coli* strains of animal husbandry origin exhibiting rates as high as 60% resistance [61]. These increased rates are underpinned by historical use of tetracyclines as growth promoters and through ongoing use in mass treatment plans instead of targeting sick animals only.

Recent research highlights that the emergence and fixation of each resistance mechanism was dependent on the generation of tetracycline routinely used at the time. Efflux emerged as the dominant mechanism of tetracycline resistance following introduction of first-generation tetracyclines. The use of second-generation tetracyclines to circumvent efflux-mediated resistance resulted in emergence of RPPs. Whilst the more ongoing emergence of TDases was linked to the use of third-generation tetracycline [23]. This landmark study explains why many bacteria will contain multiple tetracycline resistance genes, with the selection of each individual mechanism driven by associated benefits with resistance towards each generation of tetracycline.

The spread of resistance against all generations of tetracyclines is driven by the transmissibility of resistance genes through horizontal transfer. Widespread resistance to first-generation tetracyclines is largely driven by the transmissibility of the tetracycline-specific efflux pumps with *tetA*, *tetB*, *tetC*, *tetK* and *tetL* disseminated through incorporation into integrons, transposons and conjugative plasmids [62]. The RND-type MDR efflux pumps are primarily chromosomal structures conferring low-level resistance to multiple antibiotics, including all tetracycline classes. However, recent studies have revealed the presence of RND-efflux pump gene clusters on transmissible plasmids. For example, an IncFIA plasmid identified in pan-resistant *K. pneumoniae* isolates conferred a 4–32-fold increase in tigecycline, quinolones, cephalosporins and aminoglycoside MIC when conjugated into *E. coli*, *Salmonella* Typhimurium and a laboratory *K. pneumoniae* isolate [63]. It was later found to be distributed in sequences from GenBank amongst *Enterobacteriaceae* and *Pseudomonas sp*., highlighting a need to understand the contribution of these systems to widespread MDR [63]. Worryingly, TDases (TetX) have been identified on highly transferable plasmids which can disseminate amongst *Enterobacteriaceae* [50, 55]. This paints a morbid scenario in which the emergence and spread of resistance is inevitable, highlighting the importance of continued antimicrobial stewardship combined with investment in the discovery of new antibiotics.

## Clinical efficacy and contraindications

Whilst tetracyclines have historically been used as broad-spectrum antimicrobials against a range of bacterial infections, extensive levels of resistance have severely limited the use of both first- and second-generation tetracyclines against these infections. In the modern era, first-generation tetracyclines are generally for veterinary use, whilst second-generation tetracyclines are used for a limited number of bacterial infections, or used as prophylaxis to prevent infections establishing in high-risk individuals [57, 64]. Doxycycline is typically used for upper respiratory tract infections caused by *Streptococcus pneumoniae/Haemophilus influenzae* and atypical pneumonias caused by *Chlamydia pneumoniae, Mycoplasma pneumoniae, Legionella pneumophila* [65–67]. Tick-borne infections, such as Rocky Mountain Spotted Fever, typhus fever, Q pox and Rickettsialpox, are also treated by second-generation tetracyclines alongside uncommon infections caused by *Chlamydia spp*., *Yersinia spp*. and *Borrelia spp*. (Lyme disease) [68]. The uncommon nature of these pathogens means they have had limited exposure to tetracyclines; thus, the burden of resistance is decreased in comparison to frequent offenders, such as *E. coli* and *Salmonella spp*. Whilst bacterial treatment has been hampered by resistance development, tetracycline resistance has not been reported in parasitic infections, enabling unhindered use against susceptible eukaryotic pathogens [69]. Generally, tetracyclines are used for prophylaxis or in combination with first-line anti-parasite infection treatments. For example, doxycycline is used as an adjuvant alongside quinine for treatment of the malarial parasite *Plasmodium falciparum* [69, 70]. Quinine promotes haem accumulation in the parasite rapidly reducing the parasite load in the bloodstream, whilst inhibition of protein synthesis in the apicoplast by doxycycline targets residual parasites which have resisted the effects of quinine [2, 71]. Use of tetracycline as prophylaxis requires taking the antibiotic before and after visiting a risk area with the anti-malarial property of tetracycline inhibiting infection establishment [70]. Similarly, tetracyclines can be used as adjuvants in the treatment of amoeba parasites (*Entamoeba histolytica*) and other intestinal flagellates, such as *Dientamoeba fragilis* or *Balantidium coli;* however, the use of tetracycline in these cases is rare [72–74].

Tetracycline treatment duration and dosage vary depending on the infection. Patients with upper respiratory tract infections take doxycycline for 7–14 days with a 200 mg oral dose administered on the first day, followed by 100 mg daily [75]. In comparison, use for malaria prophylaxis requires tetracycline to be taken for the duration of a visit, followed by an additional 4 weeks with a daily dose of 50–100 mg, whilst treatment of balantidiasis (*B. coli*) is up to 10 days at 500 mg per day [70, 74]. It is critical to follow the advised course to ensure effective treatment, minimize resistance development and reduce the occurrence of unnecessary side effects. The list of side effects associated with tetracyclines is extensive and specific to the tetracycline type. Common side effects to the entire family of tetracyclines include nausea, vomiting, diarrhoea, photosensitivity, angioedema and rashes. Rare and more serious side effects include hepatic disorders, idiopathic intracranial hypertension, pericarditis, eosinophilia, haemolytic anaemia, dental hypoplasia, neutropenia, pancreatitis, renal impairment, Stevens–Johnson syndrome, oesophagitis and oesophageal ulcerations when administered orally, pseudomembranous enterocolitis and Henoch–Schönlein purpura. This extensive range of side-effects must be taken into consideration before prescribing tetracyclines and likely contributes to the decline in use as broad-spectrum antimicrobials alongside resistance development. The third-generation tetracyclines, such as tigecycline, are generally associated with a more complex safety profile, with the incidence of rare and serious side effects more frequent with these drugs. This has been hypothesized to be due to increased affinity of tigecycline for the mitochondrial ribosome, with lower doses typically recommended when compared to doxycycline [76]. Combined with antimicrobial stewardship requirements, this increased threat to the patient has contributed to tigecycline becoming reserved as an antibiotic of last resort when treating MDR bacteria.

Contraindications are primarily linked to the ability of tetracyclines to chelate essential metal ions. The most important contraindication is that tetracyclines should not be administered to children under 12 or in pregnant/breastfeeding women. Tetracycline chelates calcium and can cross into breastmilk, being deposited on developing bones/teeth leading to developmental issues, staining and dental hypoplasia. Similarly, tetracyclines binding to calcium reduces the conversion of prothrombin to thrombin, reducing blood coagulation after injury [77]. This means patients concurrently taking blood thinners, such as warfarin and tetracyclines, should be closely monitored for increased bleeding events. Metal-based therapies must be carefully considered when administered alongside tetracyclines as the activity of both will be diminished through chelation, with a 2–3 h gap between both therapies required to ensure optimal activity. In patients with renal impairment, tetracyclines can exacerbate symptoms. Although second-generation tetracyclines, doxycycline and minocycline are primarily excreted through the gastrointestinal tract, making them safer options for patients with renal problems. Whilst concomitant administration alongside retinoids is associated with the development of idiopathic intracranial hypertension by blocking cerebrospinal fluid (CSF) reabsorption into blood. Detailed information on clinical uses, side effects and contraindications specific to each tetracycline can be found at NICE.org.uk and clevelandclinic.org.

## Future perspectives and questions

Whilst tetracyclines are one of the most extensively used antibiotics in human history, there are still outstanding questions surrounding their use in human treatment and resistance development. Tetracyclines have been reported to induce hepatotoxicity by targeting the mitochondria of liver cells, leading to accumulation of fatty acids which disturb liver function [78, 79]. Why is the liver specifically targeted in this manner, whilst other mitochondrion-rich cell types seemingly escape these toxic effects? Similarly, tetracyclines cause a myriad of side effects; how often does this necessitate withdrawal of treatment and does this vary for each specific tetracycline? [80] or is this associated with the use of this drug as a last-chance antimicrobial, whereby patients are already significantly sicker in comparison to other tetracyclines.

In terms of resistance, can we resensitize specific bacteria to the effects of first- and second-generation tetracyclines? Identification and use of efflux inhibitors could resensitize infections to first-generation tetracyclines. Recent screens of clinically approved drugs have identified a number of efflux inhibitors which could be administered alongside tetracyclines, enabling use of these drugs against targeted organisms [81–84]. Whilst the compounds are clinically approved, they are usually used for alternative conditions and have yet to be approved for use in bacterial infections. However, since the compounds are clinically approved for other conditions, the drug pathway from discovery to implementation could be shortened. Similarly, can we selectively inhibit the activity of RPPs to enable wider utilization of second-generation tetracyclines? RPPs dislodging tetracycline from the ribosome requires GTP hydrolysis [32]. Blocking GTP hydrolysis or using nonhydrolyzable GTP analogues would inhibit the activity of RPPs, enabling clinicians to reclaim use of doxycycline and minocycline for conditions where high incidences of resistance limit use of these drugs. Given that GTP hydrolysis is essential for a number of essential processes, such as phagolysosome maturation, discovery of drugs which selectively target RPP GTP hydrolysis without off-site toxin effects will likely be difficult [85]. Of current clinical concern is the emergence of transmissible TDases which can degrade all current generations of tetracyclines. Whilst tigecycline resistance driven by TDases is rare in clinical isolates, the discovery that transmissible *tetX*-containing plasmids have been identified in gut microbiome isolates and opportunistic pathogens means that increasing exposure to third-generation tetracyclines will eventually drive resistance acquisition [47]. This would reduce clinical applications in a similar manner to first- and second-generation tetracyclines, with loss of tigecycline as a last-resort antimicrobial particularly worrying. Open questions surrounding this are (i) How transmissible are the identified TDase-encoding plasmids between clinically relevant bacteria? and (ii) How long will responsible stewardship of tigecycline prevent spread of resistance? Whilst the development of these third-generation tetracyclines was a timely intervention in an increasingly bleak antimicrobial development pipeline, does the emergence of these mobile TDases mean they are doomed to fail like their precursors? Increased monitoring akin to the T.E.S.T. study referenced in this profile are required to ensure continued effectiveness of tigecycline as an antimicrobial of last resort.

## Abbreviations

aa: amino acid
AMR: antimicrobial resistance
CSF: cerebrospinal fluid
EF-G: elongation factor G
EF-Tu: elongation factor Tu
ESBL: extended spectrum beta-lactamase producing
EU: European Union
FDA: Food and Drug Administration
MDR: multidrug-resistance
MFS: Major Facilitator Superfamily
RND: Resistance-Nodulation-Cell-Division
RPP: ribosomal protection protein
TDases: tetracycline destructases
T.E.S.T: Tigecycline Evaluation Surveillance Trial
WHO: World Health Organization
